# Supplementary material for: Identifying key areas of post-secondary student stress: Principal component analysis of the Post-Secondary Student Stressors Index (PSSI)
Source: PLOS Ment Health. 2025 Jan 3;2(1):e0000061. doi: 10.1371/journal.pmen.0000061 (PMC12798379; doi:10.1371/journal.pmen.0000061)
Supplement: Attachment — Submitted filename: Response to reviewers.docx [file pmen.0000061.s001.docx]

Response to reviewers

The authors thank the journal for their detailed review of the manuscript. We have now addressed the following concerns.

1. Please complete all items on the Human Participants Research Checklist that are relevant for your submission, by following this link: http://journals.plos.org/plosone/s/file?id=dc11/PLOSOne_Human_Subjects_Research_Checklist.docx. There may be overlap between the checklist items and other queries listed here; please address any duplicated queries both in your response email and on the checklist itself. Upload the completed Human Participants Research checklist as file type “Other” when you re-submit your manuscript. This document is for internal journal use only and will not be published if your article is accepted. The requested information will help us to assess whether your submission complies with PLOS’s policies and adheres to applicable reporting standards. Note that your manuscript may be rejected if you provide incomplete or inadequate responses to the checklist questions and that changing the ‘Section/Category’ of your article does not affect this requirement.

Response: The checklist items were addressed within the manuscript. We have also attached a completed checklist as requested.

2. Your study includes live participants, but you have not included an Ethics Statement. Please include an Ethics Statement subsection to your Materials and Methods, to include:

   i) The name(s) of the Institutional Review Board(s) or Ethics Committee(s)

   ii) The approval number(s), or a statement that approval was granted by the named board(s) 

   iii) (for human participants or donors) - A statement that formal consent was obtained (must state whether verbal/written) OR the reason consent was not obtained (e.g., anonymity).

Response: An ethics statement was included in the methods section of the manuscript. We have also clarified that consent was obtained.

Thank you.

Shay-Lee Bolton PhD
