# Supplementary material for: Identifying key areas of post-secondary student stress: Principal component analysis of the Post-Secondary Student Stressors Index (PSSI)
Source: PLOS Ment Health. 2025 Jan 3;2(1):e0000061. doi: 10.1371/journal.pmen.0000061 (PMC12798379; doi:10.1371/journal.pmen.0000061)
Supplement: Attachment — Submitted filename: Response to Reviews_BL 5Nov2024_sb.docx [file pmen.0000061.s002.docx]

Dear Ms. Charlene Sunkel & Dr. Rochelle Burgess,

Re: **Identifying key areas of post-secondary student stress: Model reduction using principal component analysis in the Post-Secondary Student Stressors Index (PSSI)**

*[title modified to: Identifying key areas of post-secondary student stress: Principal component analysis of the Post-Secondary Student Stressors Index (PSSI)****]***

Thank you for your thoughtful review of our manuscript and for providing the opportunity to revise and resubmit. We are happy to provide the attached revised version of the manuscript with responses to the Reviewers’ comments illustrated below. Please see our detailed responses below.

**Editorial comments:** 

**1. Thank you for your response to our query. You may please add Dr Maher in the acknowledgements. Thank you for your attention.**

*Response: Thank you for your suggestion. We have added the following sentence to the Acknowledgements section of the manuscript “The authors would like to thank Dr. Alyson Mahar for her contribution to this work”(see page 16).*

**2. Please send a completed 'Competing Interests' statement, including any COIs declared by your co-authors. If you have no competing interests to declare, please state "The authors have declared that no competing interests exist". Otherwise please declare all competing interests beginning with the statement "I have read the journal's policy and the authors of this manuscript have the following competing interests:"**

*Response: A ‘Competing Interests’ statement has been added to the submission of the manuscript on the PLOS Mental Health Portal to reflect all COIs declared by co-authors of the manuscript. The ‘Competing Interests’ statement includes the aforementioned statement “I have read the journal’s policy and the authors of this manuscript have the following competing interests:”*

**3. Please provide a/amend your detailed Financial Disclosure statement. This is published with the article. It must therefore be completed in full sentences and contain the exact wording you wish to be published.**

****Please only choose the relevant sentences from below****

**1. Please clarify all sources of funding (financial or material support) for your study. List the grants (with grant number) or organizations (with url) that supported your study, including funding received from your institution. 
2. State the initials, alongside each funding source, of each author to receive each grant.
3. State what role the funders took in the study. If the funders had no role in your study, please state: “The funders had no role in study design, data collection and analysis, decision to publish, or preparation of the manuscript.”
4. If any authors received a salary from any of your funders, please state which authors and which funders.**

**If you did not receive any funding for this study, please simply state: “The authors received no specific funding for this work.”**

*Response: Thank you for your suggestion. A detailed Financial Disclosure statement has been completed on the PLOS Mental Health portal for submission with the manuscript, including full sentences and the exact wording we wish to be published. All sources of funding, including grants and institutional funding, have been clarified with respect to authors and funders.*

**4. "H2020 295 Approval Cert.pdf" is currently uploaded as an 'Supporting Information' file type. Please ensure that it is uploaded as 'Other'.**

*Response: “H2020 295 Approval Cert.pdf” has been uploaded as ‘Other’ to the PLOS Mental Health portal.*

**5. We note that you have indicated that there are restrictions to data sharing for this study. For studies involving human research participant data or other sensitive data, we encourage authors to share de-identified or anonymized data. However, when data cannot be publicly shared for ethical reasons, we allow authors to make their data sets available upon request. For information on unacceptable data access restrictions, please see http://journals.plos.org/plosone/s/data-availability#loc-unacceptable-data-access-restrictions. 
 
 Before we proceed with your manuscript, please address the following prompts:
 
 a) If there are ethical or legal restrictions on sharing a de-identified data set, please explain them in detail (e.g., data contain potentially identifying or sensitive patient information, data are owned by a third-party organization, etc.) and who has imposed them (e.g., a Research Ethics Committee or Institutional Review Board, etc.). Please also provide contact information for a data access committee, ethics committee, or other institutional body to which data requests may be sent.
 
 b) If there are no restrictions, please upload the minimal anonymized data set necessary to replicate your study findings to a stable, public repository and provide us with the relevant URLs, DOIs, or accession numbers. Please see http://www.bmj.com/content/340/bmj.c181.long for guidelines on how to de-identify and prepare clinical data for publication. For a list of recommended repositories, please see https://journals.plos.org/plosone/s/recommended-repositories. You also have the option of uploading the data as Supporting Information files, but we would recommend depositing data directly to a data repository if possible.
 
Please update your Data Availability statement in the submission form accordingly.**

*Response: Due to ethical restrictions on data sharing for this study, data has been indicated as available upon request in the Data Availability statement uploaded to the PLOS Mental Health portal. The data restrictions have been explained in detail and contact information has been provided.*

**General guiding points from the Authors in response to Reviewer comments:**

*A number of comments from Reviewer 1 and Reviewer 3 drew our attention to a miscommunication conveyed by this work in the initial submission. To provide clairification, the primary aim of this work was not to reduce or simplify the number of items on the PSSI using principal component analysis. Rather, the primary aim of this work was to formulate a clearer conceptualization of areas of stress (“components” of stress) for post-secondary students in a novel context (Manitoba) through a rigorous statistical approach, that could be used in other contexts. Given this overarching goal, our study aimed to improve utilization of the PSSI in two ways: 1) principal component analysis was utilized in the creation of subcomponents (domains) of stress as defined by the PSSI dataset in this population, and 2) inclusion of both frequency and severity of each stressor produced a more accurate depiction of post-secondary student stress. The reorganization of the PSSI using a statistically-founded framework will allow for its use in future research.*

*As such, the authors reframed and clarified the rationale and aims of this work significantly to align with this goal.* *It is therefore the case that many of the reviewer comments regarding study rationale, theoretical aspects, and model reduction were addressed substantially through the improved clarity of these goals. In addition, the title of the present work has been revised to reflect the study’s aims, as has some of the terminology used throughout the manuscript.*

**Reviewer 1 Comments:**

**1. Lack of theoretical aspects**

*Response: Thank you for highlighting this limitation of the Introduction section. A theoretical framework behind post-secondary student stress has been added to the Introduction – the Stress Sensitization Model (see page 3). The study rationale featured in the Introduction section has been augmented to explore the reasons why we felt this study was important – including the unique stress experiences of post-secondary students compared to the general adult population (page 3), the importance of evaluating different types of stress (pages 3-4), and the limitations of previous work done in the area of post-secondary student stress (pages 5-6; also see general guiding points above).*

**2. Lack of recent scientific study.**

*Response: The study references have been revised and updated to reflect more recent scientific literature throughout the paper.*

**3. Rationale of current study is need.**

*Response: As per comment 1, we have revised the Introduction of the current study with the inclusion of a theoretical background (the Stress Sensitization Model; see page 3). Additionally, the study rationale has been expanded throughout the Introduction section, with emphasis placed on the unique stress experiences of post-secondary students (page 3), the importance of assessing different types of stress (pages 3-4), and the limitations of prior work done in this research area (pages 5-6; also see general guiding points above).*

**4. Scope of current study should be included.**

*Response: As per above, we have strengthened the rationale for the current study using a theoretical framework (see page 3). We have also clarified the current study aims and scope (see pages 5-6). We have modified the previous study aims to increase clarity and better reflect the stated goals of the research (also see general guiding points above).*

**5. Significance of current study is lacking.**

*Response: Thank you for highlighting this point. We have now enhanced the clarity of the significance of the study (see page 3). The significance of the study is exemplified by the relationship between chronic stress and mental health problems. Moreover, the study explains that the understanding of student stress is helpful in the designing of interventions and supports (see page 6). The use of a theoretical framework behind post-secondary student stress has also clarified the importance of the work (see page 3). The justification for this research has been augmented in the Introduction section using research-based evidence. Special attention is drawn to two limitations of prior work – a need to build clear domains of stress to better address concerns of post-secondary students, and a lack of integration of frequency and severity of stressors in prior work with PSSI data (see pages 5-6; also see general comments above).*

**6. Need to mention the design of the current study.**

*Response: Thank you for this comment. The design of the current study – a secondary data analysis of a longitudinal cohort – has been explicitly referenced in the Methods section of the manuscript on page 6.*

**7. Lack of information regarding study area.**
*Response: Thank you for pointing out this lack of clarity. As per comment 6, study area (i.e., the Manitoban subsample) and the novelty/importance of this study area has been clarified in the Introduction and Methods sections (see pages 5-6). This revision clarifies some confusion regarding study area.*

**8. Need to add the Cronbach's alpha of each scale.**
*Response: We appreciate the Reviewer’s comment. The Cronbach’s alpha of the Post-Secondary Student Stressors Index (PSSI) was not previously reported and thus we were unable to add this value into the Methods section. Reliability of the PSSI was evaluated, however, in the Manitoba sample and supplemented in the Results section (see page 13). Due to other reviewer comments, we have removed a number of additional scales from the manuscript, and therefore alpha for these scales is no longer applicable.*

**9. Poor future directions.**
*Response: The Conclusion has been expanded-upon and strengthened with research-based evidence and more conceptually-clear indications for future directions (see pages 15-16). The accessibility and utility of the revised structure as a statistically-founded, Manitoba-specific version of the PSSI has been highlighted in the Conclusion (see pages 14-15). The structure’s identification of specific, priority areas of stress for post-secondary students, based on the inclusion of both frequency and severity of stressors, will be a valuable resource for policymakers and campus resource allocation. Future directions will include the validation of this structure in additional Canadian contexts.*

**10. Limitations should be clear.**
*Response: The limitations listed in the Discussion section have been clarified in their wording and enhanced through the addition of evidence-based explanations and references (see page 15).*

**11. Lack of conclusion.**
*Response: With reference to earlier comments (comments 1, 3, 4, 5) provided by Reviewer 1, the strengthened rationale in the Introduction section is revisited in the Discussion section (see pages 13-16). The enhanced theoretical framework and rationale for the study facilitate a stronger conclusion to the study. The conclusion of the Discussion section has also been enhanced through the incorporation of the utility of the revised structure in future research (per comment 9; see pages 15-16). This includes the significance of the revised structure as a mental health resource on university campuses. This novel framework of student stressors, based on different subcomponents of stress, can be utilized towards targeted intervention approaches and delineation of resource allocation (also see general comments above).*

**12. The relevance of model reduction using principal component analysis is lacking.**
*Response: Thank you for highlighting this major limitation inherent in the previous rationale and aims for this study. As per earlier comments by Reviewer 1, the rationale for the study was strengthened (see pages 3-6) and a theoretical framework (see page 3) was supplemented to explain why authors chose to examine the underlying components of the PSSI. These revisions will allow for more clarity surrounding the purpose of the study. Our study rationale and aims have been modified to reflect prior comments (see pages 5-6). We have clarified within the manuscript that rather than an aim of model reduction of the PSSI using principal component analysis, we are instead highlighting that the PSSI was examined using principal component analysis in order to better understand the priority components of stress for post-secondary students (see pages 5-6). The PCA – a methodologically-founded framework – was utilized in order to group together items of the PSSI in a coherent, statistically-driven fashion (also see general comments above).*

**Reviewer 2 Comments:**

**1. Your manuscript is methodologically sound and makes a significant contribution to the field. Conduct a thorough proofreading to catch any minor typographical or grammatical errors that may have been overlooked. This will help ensure the manuscript meets the high standards expected by PLOS Mental Health.**

*Response: Thank you for your review of the manuscript and your valuable feedback. A thorough proofreading of the manuscript has been conducted to ensure the absence of any typographical and grammatical errors.*

**Reviewer 3 Comments:**

**This work left me with far more questions than answers. I’m listing my questions here roughly in order from those I feel are most significant to those that are less important (and might even become non-issues, depending upon how the earlier questions are resolved).

1. What is the intent of this work? The title and some content in the introduction suggest the primary aim is to reduce the number of items on the PSSI, but the methodology that is implemented is poorly suited to this goal. As a result, the work ends up suggesting that the domains underlying the PSSI should be revised.**

*Response: Thank you for this important question. As per comments above (also see general comments), the title and certain portions of the work (including in the Introduction) have been revised to reflect the true primary aim of this work – which was not the reduction of the number of items on the PSSI (see pages 3-5). Rather, the primary aim of this work was to utilize the PSSI in a novel way – to delineate specific areas/components of student stress. This work utilizes a different sample than that which was previously utilized in examining the PSSI. Thus, in our sample, the domains look different. We also utilized a different methodology (PCA) and implemented the use of both frequency and severity rankings from Likert scales. Prior evaluations of the PSSI did not utilize the full scale to assess both severity and frequency of stressors, but rather assessed stressors on a “yes” or “no” basis in terms of frequency. It is important to note that previous work grouped items into domains based on face validity, following administration of the questionnaire. The current study asserts that the use of PCA to reorganize the PSSI in this context will result in the construction of a novel way to conceptualize components of post-secondary student stress (see pages 5-6).*

**2. Is there confidence in the measurement model underlying the PPSI-46? If so, a confirmatory methodology (such as formative structural equation modeling) could be used to identify which items to drop. This approach would enable direct evaluation of the impact of these changes on the overall goodness of fit, and would avoid the problem of the authors’ measurement model yielding different domains than have been previously reported in the literature.**

*Response: Thank you for your suggestion. The authors would like to reiterate that the goal of the present work was not to reduce the number of items of the PSSI, but to understand the different components of stress and the full range of student stress experiences (by utilizing the Likert scale). As such, confirmatory factor analysis of the previously constructed model for the PSSI would not be sufficient for our aims. (also see general comments above)*

**3. What should readers conclude from the different domains that were found in previous analyses of the PSSI-46 (and the Brief-PSSI) and the findings reported in the present work? Do the discrepant findings result from differences in the sample, or from scoring differences (using Likert scales instead of binary variables to score the PSSI)? Which domains provide a better description of the PSSI measurement model, and what evidence supports this conclusion?**

*Response: The domains that were reported in the present work did differ from the domains reported in previous analyses of the PSSI. These findings were to be expected, considering that this study utilizes a different sample than that which was utilized previously. Authors also implemented scoring differences (using Likert scales instead of binary variables to score the PSSI). This constitutes a different methodology by implementing use of both frequency and severity rankings. These methods may give more measurement variance and therefore findings are not surprisingly discrepant from prior work. In terms of which model is better, this is difficult to assert as the methodologies used are different. At this time, the authors would leave it to the readers to decide their preferred model based on the strength of the work. We have included in our rationale and conclusions how we feel that this manuscript has improved upon prior work.*

**4. Which methods were used to identify the five domains in the PSSI-46, and how do these differ from the methods in the present work? What methods were used for factor extraction and rotation during development of the PSSI-46? What factor loading cutoff was used to retain items? What is the available psychometric evidence for the reliability and validity of the PSSI-46? Most of these details seem to be absent from the references cited by the authors but are important for contextualizing the present work. For example, researchers often use principal component analysis (PCA) to extract factors but describe this as exploratory factory analysis. Was this the approach used for the PSSI-46? If so, the lengthy justification for using PCA in the present work seems inappropriate.**

*Response: Extensive details regarding the development and complete psychometric evaluation including the collection of content, response processes, relations to other variables, and internal structure evidence for validity, in addition to test-retest reliability and related sensitivity analyses are reported at length in Linden and Stuart 2019 (https://bmcpublichealth.biomedcentral.com/articles/10.1186/s12889-019-7472-z).*

*The original five domains of stress in the PSSI were determined on a* ***conceptual*** *level through the qualitative component of the sequential mixed methods research project conducted to initially create the tool. Stressors were categorically ‘sorted’ by participating students, with an overarching ‘theme’ or domain assigned. The original “factor analysis” conducted to assess the internal structure of the tool was a principal components analysis (PCA) with a Kaiser normalization and varimax rotation (KMO statistic >0.7 and Bartlett’s test for sphericity p<0.001). The factor loading cut-off was set to 0.3, per convention.*

*Importantly, as noted in Linden and Stuart 2020, “The PSSI was designed to be an index, rather than a scale so individual stress items were conceptualized as causal indicators (e.g., “causes” of stress), rather than effect indicators (e.g., “effects” of stress), as would be the case with a scale. …[we sought] to determine whether the PSSI had the dimensionality of an index, as hypothesized.” Thus, the goal of the original validation of the tool was not to reduce the number of items present. The results of this analysis, per Linden and Stuart 2019, “… supported our hypothesis that the PSSI would take the internal structure of an index, rather than a scale. […] no clear groups of items emerged as viable subscales, making it appropriate to treat each item as an individual causal indicator of our underlying construct of post-secondary student stress.”*

*With the benefit of hindsight and an additional ~7 years of conducting psychometric research, I (BL) can now recognize that the use of PCA was not the most appropriate choice given my analytic goals at the time. Given that I had no desire to reduce the number of items included in the instrument, an exploratory (or “common”) factor analysis maybe have been more appropriate, in addition to the use of an oblique rotation. That being said, as stated above, the goal at the time was not to “redefine” or identify the underlying latent constructs of interest (i.e., domains of stress) as these had been conceptually determined a priori.*

*In addition to the PCA demonstrating the PSSI to have the internal structure of an index, the tool also demonstrated strong content and response processes evidence as determined through the use of a modified Delphi method and cognitive interviewing, respectively, in addition to strong relations to other variables evidence in its relationship to like instruments including the Perceived Stress Scale (10-item), Kessler Psychological Distress Scale (K10), and Connor-Davidson Resiliency Scale (CD-RISC-10). Test-retest reliability was evaluated and shown to be strong across all subgroup sensitivity analyses. Internal consistency reliability (i.e., Cronbach’s alpha) was not assessed given its inappropriate usage with indices (see Streiner 2003 https://pubmed.ncbi.nlm.nih.gov/12763696/).*

*In addition to this initial validation work, a Confirmatory Factor Analysis was also conducted on the original PSSI using a cross-national sample of students attending post-secondary school across Canada (Linden and Stuart 2022,* [*doi.org/10.1371/journal.pone.0278897*](https://doi.org/10.1371/journal.pone.0278897)*). In this more recent study, we estimated MIMIC models (structural equation modelling) to further investigate the internal structure of the tool, this time specifying the five domains of stress as our latent variables and age and gender as predictors. The model was ultimately significant with strong factor loadings. Goodness-of-fit statistics were acceptable, but could have been stronger perhaps with some revision to the tool. However, recognizing that each institution’s student landscape and social context may differ, it is to be expected that some institutions may see some stressors perform as more salient on their campus compared to others.*

**5. The authors argue for the need to assess “different types of stress” (line 49) and criticize other works because they evaluate “only one dimension of stress” (line 54). What is the justification for assessing multiple types of stress? I can think of several reasons but am interested in the authors’ motivations here. If it is indeed important to measure several dimensions of stress, why do the authors derive a 10-domain measure of stress but only report statistics relating to a single, overall stress measure?**

*Response: Thank you for your comment. We agree with the reviewer that there are multiple reasons to assess several dimensions of stress. Literature that details justification for assessing multiple types of stress has been added to the Introduction section of the manuscript (see page 3). One of the most important reasons to assess different types of stress in post-secondary students is because student stress is a multidimensional concept. Although some stressors may co-occur, others may differ based on individual characteristics and values. One of the ways in which authors aimed to measure different dimensions of stress was through the assessment of both the severity of stress experienced and frequency with which each stressor occurred. A clearer understanding of the individualized nature of stress is important in the development of focused resources that are personalised to post-secondary student experiences. As such, the authors agree with the reviewer’s point that a single overall statistic of stress does not align with the goals of the paper. As such, we have replaced Table 3 with a table that displays reliability coefficients for each of the 10 components of the PSSI measure (see page 13). (also see general comments above)*

**6. What evidence is there to support the reliability of the PSSI-40? Internal validity isn’t reported (although lines 292-295 implies that it is). Similarly, lines 295-296 suggest test-retest reliability was calculated, but no details about this are provided in the paper.**
*Response: Given our response to prior comments, the internal validity of the PSSI-40 is no longer warranted. We have removed all reference to the revised structure (PSSI-40) of the overall scale.*

**7. Should readers be surprised that the PPSI-40 “achieved similar psychometric properties in comparison with the PSSI-46” (lines 275-276), given that only total scores were used and the two instruments share 40 items?**
*Response: Given our response to previous comments, the comparison of the PSSI-40 and PSSI-46 has been removed from the manuscript.*

**8. How do the authors conceptualize “diversity”? Does this term – which is mentioned several times – refer to diversity in geographic location, socioeconomic status, first generation status, gender, race, ethnicity, native language, or … ? Lines 80-81 seem to imply that the actual concern is not really diversity, but is instead that findings derived from administering the PSSI in other geographic locations may not be generalizable to students in Manitoba. If so, this should be stated explicitly and comparisons should be made between Manitoba findings and findings from other locations. Otherwise, more information should be provided about what "diversity" means in the present context. This could require revising Table 1 to characterize the diversity represented in the sample (to the extent that this information is available). One could also argue that descriptive statistics should be reported for PSSI (and PSS-10, K10, and CD-RISC) scores disaggregated by the diversity characteristic(s) of interest.**

*Response: The term “diversity” was conceptualized as the idea that there exist different types of stress for socioculturally-diverse persons (see pages 3-4). Stress can also differ by study population and sample demographics (see pages 3-4). The Reviewer’s point was well-taken. The authors agree that the use of the word “diversity” was not the best fit for describing different populations that were implied. The authors largely used “diversity” as an umbrella term to describe a number of different diversities that have been identified in Reviewer 3’s comment. We’ve largely removed the term and replaced it with the term “differences”, when applicable. Reviewer 3 is correct in their assessment that the concerns with diversity are not a driving factor in the development of a revised structure of the PSSI. Rather, it is more so the case that the PSSI has not previously been validated in a different provincial sample, and therefore components of stress may not be applicable to Manitoba post-secondary students. The authors have made this statement more explicit in the present work, and it is mentioned more than once throughout the paper (see pages 5, 15).*

**9. How many duplicates were in the original data, requiring the application of the steps described in lines 124-129? Presumably there would be no concern regarding duplicates if only the T1 data were examined. What sample size would have resulted from including only T1 data, and why did the authors choose instead to include data from other waves? Might this practice have added undesired variance (and, perhaps, systematic biases of some sort) to the data? What might lead a student to miss T1 but provide responses to T2 or T3?**

*Response: An inherent challenge of the data included several cases of duplicate participant IDs, where different participants were assigned the same ‘unique’ respondent ID. Authors constructed a list of duplicates across timepoints. For these duplicates, we matched individuals based on five characteristics (ID, sex, birth year, international student, first generation student). For cases in which individuals with the same ID did not match on all five characteristics across duplicate timepoints, we assumed that these were different individuals and assigned a unique ID. The original sample size consisted of 3647 participants. There were 76 duplicates in the original data. Following the sorting of duplicate cases, there was a total sample size of 2856 post-secondary students across all three timepoints. It is true that these practices constituted a limitation of the work, as authors do not know why students missed some timepoints. We added this limitation to the Discussion section in the manuscript (see page 15). What might lead a student to miss T1 but provide responses to T2 or T3? – this is an excellent empirical question. We can hypothesize reasons why students might miss one of the timepoints – for example, students were stressed, workload prevented completion, lack of interest in participating, etc. As such, inclusion of a statement of limitation is appropriate in this case. The idea behind utilizing the first timepoint for each individual across the 3 data collection waves was to maximize the amount of data that was used. By increasing sample size, we attempted to ensure the stability of findings across a broader sample of individuals.*

**10. Is the reduction in items from 46 to 40 meaningful from the perspective of the respondent? What evidence or logic supports this position? Is either version superior to the Brief-PSSI (which contains only 10 items), and why?**

*Response: As highlighted in our response to earlier comments, the primary aim of the present work has been revised. Rather than the primary aim encompassing the reduction of the PSSI model to include fewer items, the primary aim is to derive components of stressors for post-secondary students. Founded in statistical method, and with the use of a unique sample, these components of stress will help to determine priority areas of stress for post-secondary students. To reflect this change in rationale, all language referring to “PSSI-46” and “PSSI-40” has been removed. The authors would argue that the reduction in items from 46 to 40 is not particularly meaningful from the perspective of the respondent, and thus have not made this conclusion. The authors also acknowledge the existence of the Brief-PSSI (which contains 14 items), however since the goal of the study is not item reduction, this scale is not viewed as relevant to the current work. We are not intending to compare the component approach as superior or inferior to the Brief-PSSI, as the aims of these two works differ significantly.*

**11. What are the samples sizes for each wave in Manitoba? Lines 107-108 seem to report sample sizes for all of Canada.**

*Response: Thank you for this suggestion. The sample sizes for each wave in Manitoba (Central Canada region) has been supplemented in the first paragraph of the Methods section, following the listing of the sample sizes for all of Canada (see page 6).*

We appreciate the helpful suggestions and comments from these Reviewers and the Executive Editor. We hope that our manuscript will now be considered for full acceptance for publication in PLOS Mental Health.

Sincerely,

Shay-Lee Bolton
